# Induced abortion and effecting factors of ever married women in the Southeast Anatolian Project Region, Turkey: a cross sectional study

**DOI:** 10.1186/1471-2458-4-65

**Published:** 2004-12-22

**Authors:** Ali Ihsan Bozkurt, Birgul Özcirpici, Servet Ozgur, Saime Sahinoz, Turgut Sahinoz, Gunay Saka, Ali Ceylan, Ersen Ilcin, Hamit Acemoglu, Yilmaz Palanci, Feridun Akkafa, Mucide Ak

**Affiliations:** 1 Department of Public Health, Faculty of Medicine, Pamukkale University, Denizli, Turkey; 2 Department of Public Health, Faculty of Medicine, Gaziantep University, Gaziantep, Turkey; 3 Medical Directorate of Gaziantep Province, Gaziantep, Turkey; 4 Department of Public Health, Faculty of Medicine, Dicle University, Diyarbakır, Turkey; 5 Department of Medical Biology, Faculty of Medicine, Harran University, Şanlıurfa, Turkey; 6 Department of Parasitology, Faculty of Medicine, Ege University, İzmir, Turkey

## Abstract

**Background:**

Nearly 10% of the population of Turkey lives in the Southeast Anatolian Project (SEAP) region. The population growth rate and the rate of unintended pregnancies are high and family planning services are insufficient in this region. Lifetime induced abortion rate is also high in this region.

Public health problems of the SEAP region were investigated in the "SEAP Public Health Project" in 2001 and 2002. As it is one of the most important health problems of the women living in this region; induced abortion was also investigated in this project.

**Methods:**

An optimumsample size representing the rural and urban area of the region (n = 1150) was chosen by the State Institute of Statistics by a sampling method proportional to size. 1126 of the area's 1150 houses have been visited and data about induced abortions have been obtained by applying a questionnaire to 1491 ever married women who live in the region.

**Results:**

It has been found that 9.0% of these women who had at least one pregnancy in their life had at least one induced abortion. The lifetime induced abortion per 100 pregnancies was found to be 2.45. The primary reason given for induced abortions was "wanting no more children" (64.6%). Lifetime induced abortions were 5.3 times greater with women using a family planning method than women not using family planning methods. Lifetime induced abortions were 4.1 times greater with unemployed women than working women. Most of the women have used private doctors in order to have an induced abortion.

Although 32.29% have not yet begun to use a contraceptive method after their last induced abortion, 43.75% of the women have since started to use an effective contraceptive method. 23.96% of them have begun to use an ineffective contraceptive method.

**Conclusions:**

Induced abortion is still an important problem at the SEAP region. The results of the study remind us that unemployed women and women who have more than four children is our target group in the campaign against induced abortions. Most of the women use private doctors in order to have an induced abortion. Thus, priority must be given to educate private gynecologists with respect to induced abortion. After induced abortions, a qualified family planning consultant can be given to women and they can be secured to use a suitable contraceptive method.

## Background

Abortion is defined by World Health Organization (WHO) as a pregnancy that ends before 28^th ^week of gestation. Abortions are divided into two groups as 1) induced abortion and 2) spontaneous abortion. The spontaneous abortion rate increases when the maternal and natal care is insufficient. Induced abortions occur at the desire of the couple and an increase in induced abortion rate is a good indicator of insufficient family planning services. The aim of the family planning services is the prevention of unwanted pregnancies. Inadequate access to contraceptive methods, method failure caused by misuse of the methods and non-use of effective methods are the reasons of unwanted pregnancies, which lead women to induced abortion [1].

Induced abortions have been used as a family planning method for many years and become an important problem in women's health especially in developing countries. It is one of the main causes of death of women of reproductive age [2]. Induced abortions have many health disadvantages especially when performed in unsafe conditions. In a study it has been found out that abortion may be a risk factor for subsequent depression for a period of 8 years after pregnancy occurs [3]. In another study the mortality rate for induced abortion was found to be 5.3% and this accounted for 21.1% of the total maternal deaths for this period [4]. As it is seen from these studies, induced abortions have many health disadvantages for women and thus induced abortions should not be used as a family planning method.

In Turkey, the Population Planning Law legalized the provision of safe abortion services within ten weeks in May 1983. As a result, the facility to terminate unwanted pregnancies in safe conditions has been provided [2]. But induced abortion rates are different in the different regions of Turkey. Lifetime induced abortion rate is 26.6% for the whole of Turkey. However, this rate differs from 17.8% to 30.9% for the different regions of Turkey [2].

Nearly 10% of the population of Turkey lives in the Southeast Anatolian Project (SEAP) region. The population growth rate and the rate of unintended pregnancies are high and family planning services are insufficient in this region.

Public health problems of the SEAP region were investigated in the "SEAP Public Health Project" in 2001 and 2002. Induced abortion was one of the health problems investigated in this project.

## Methods

The Southeast Anatolian Project (SEAP) region has a population of approximately 6 million people and nearly 10% of the population of Turkey lives in this region. The population growth rate and the rate of unintended pregnancies are high and family planning services are insufficient in this region.

Public health problems of the SEAP region was investigated in the "SEAP Public Health Project" and this project was supported by the SEAP Regional Development Management of Prime Ministry Republic of Turkey and conducted by a consortium constituted by the Turkish Parasitology Association, Gaziantep University, Dicle University (in Diyarbakır province) and Harran University (in Şanlıurfa province). Induced abortion – an important problem for women – was investigated in this project in 2001 and 2002.

The population of the nine provinces in the region is 6,128,973. In order to investigate the public health problems of the region such as abortion, an optimumsample size which represents the rural and urban area of the region was determined as 6900 (d = 0.03, p = 0.04, α = 0.01). This number (6900) was divided to the average number of households (approximately 6 people live in each house in the SEAP region) and the number of houses in the sample was found to be 1150. An optimum sample size representing the rural and urban area of the region was chosen by the State Institute of Statistics by a sampling method proportional to size.

Questionnaires were prepared by the academic staff of public health departments of medical faculties of the two universities (Gaziantep and Dicle Universities). Three of the questionnaires were for individuals (the questionnaire of 5 year and older girls and women, the questionnaire of 5 year and older boys and men, and the questionnaire of 0–59 month's old children) and one of the questionnaires was about the house conditions. Before the study, the questionnaires were applied to houses that were not in the study sample as a pilot study and then checked.

A team for questionnaire application was constituted in every province and the teams were educated about the questionnaires. These teams visited all of the houses in the sample with a public health specialist (the head) and applied the questionnaires by face-to-face interview. Data about the people living in the house were obtained by the house questionnaire. Data about the demographic features of women, fertility, and features about abortion were obtained by the questionnaire from 5 year and older girls and women. Educated nurses (all of them were women) applied the questionnaire to women by face-to-face interview in a separate room.

1126 households of the area's 1150 houses participated to the survey. Households of the 24 houses were not found at home during the study. There were 1491 ever married (married, divorced or widow) women in the 1126 houses that participated in the survey.

The data were evaluated using the SPSS 5.0 and Excel programs. Chi-square, Student's t test and logistic regression analysis were used for the statistical analysis.

## Results

There were 1491 ever married (married, divorced or widow) women in the 1126 houses that participated in the survey. 1266 (84.9%) of these women had at least one pregnancy in their life.

9.0% of the women who were ever married and who had at least one pregnancy in their life have had at least one induced abortion in the past. The rate of the women who have had two or more induced abortions was found to be 3.63%. The lifetime induced abortion rates of the women who were ever married and who had at least one pregnancy in their life according to some basic factors are shown in Table 1.

**Table 1:** The lifetime induced abortion rates of the women who were ever married and who had at least one pregnancy in their life according to some basic factors

**Number of induced abortions**										
		**0**		**1**		**≥2**		**Women who have made at least one induced abortion**		

		**n**	**%**	**n**	**%**	**n**	**%**	**n**	**%**	**Total**

**Type of residence**	Rural	462	93,15	21	4,23	13	2,62	34	**6,85**	496
	Urban	690	89,61	47	6,10	33	4,29	80	**10,39**	770
Statistical result *p < 0.05										
**Age groups (year)**	15–19	38	100,00	0	0,00	0	0,00	0	**0,00**	38
	20–24	143	95,33	7	4,67	0	0,00	7	**4,67**	150
	25–29	199	93,87	11	5,19	2	0,94	13	**6,13**	212
	30–34	145	91,77	9	5,70	4	2,53	13	**8,23**	158
	35–39	177	87,62	12	5,94	13	6,44	25	**12,38**	202
	40–44	99	84,62	10	8,55	8	6,84	18	**15,38**	117
	45–49	91	82,73	10	9,09	9	8,18	19	**17,27**	110
	50+	260	93,19	9	3,23	10	3,58	19	**6,81**	279
Statistical result *p < 0,01										
**Education**	Illiteracy	753	92,85	35	4,32	23	2,84	58	**7,15**	811
	Literacy	85	89,47	4	4,21	6	6,32	10	**10,53**	95
	Graduated a primary school	248	86,71	23	8,04	15	5,24	38	**13,29**	286
	Graduated a secondary school	26	89,66	1	3,45	2	6,90	3	**10,34**	29
	Graduated a high school or higher	40	88,89	5	11.11	0	0,00	5	**11.11**	45
Statistical result *p < 0.05										
**Employment**	Unemployed	567	86,17	54	8,21	37	5,62	91	**13,83**	658
	Employed	585	96,22	14	2,30	9	1,48	23	**3,78**	608
Statistical result *p < 0.0001										
**Ethnicity**	Turkish	398	84,65	39	8,32	33	7,04	72	**15,35**	470
	Kurdish	629	94,16	28	4,19	11	1,65	39	**5,84**	668
	Arabic	93	100,00	0	0,00	0	0,00	0	**0,00**	93
	Zaza	32	91,43	1	2,86	2	5,71	3	**8,57**	35
Statistical result *p < 0.0001										
	**TOTAL**	1152	91,00	68	5,37	46	3,63	114	**9,00**	1266

*One and two or more induced abortions have been evaluated together in the statistical analyses.

The percentage of women who have made at least one lifetime induced abortion was higher in women living in urban areas (10.39%) than women living in rural areas (6.85%, p < 0.05). The percentage of lifetime induced abortion was higher in 35–49 age group (especially in 45–49 age group) than the other age groups (p < 0.01). Lifetime induced abortion rate was 7.15% in illiterate women and 10.53% in literate women and was higher among women who graduated from a primary school or higher (%12.77, p < 0.05). The percentage of women who have had at least one induced abortion was found to be higher in unemployed women and Turkish women than the other groups (p < 0.0001, Table 1).

The lifetime induced abortion rates of the women who were ever married and who had at least one pregnancy in their life according to some fertility characteristics are shown in Table 2. The age of the women at her first birth, number of still birth and spontaneous abortion did not affect the rate of lifetime induced abortion. The number of living children of the women was related to the number of lifetime induced abortion. The induced abortion rate was significantly high in women having 4 or more children (p < 0.01). A similar relationship was found between induced abortion and total number of pregnancies. Lifetime induced abortion was 12.09% among women who had five and more pregnancies and this was higher than the other groups (p < 0.001).

**Table 2:** The lifetime induced abortion rates of the women who were ever married and who had at least one pregnancy in their life according to some fertility characteristics

**Number of induced abortions**										
		**0**		**1**		**≥2**		**Women who have made at least one induced abortion**		

		**n**	**%**	**n**	**%**	**n**	**%**	**n**	**%**	**Total**

The age of women at her first birth	12–19	770	90,06	49	5,73	36	4,21	85	**9,94**	855
	20–24	299	91,72	17	5,21	10	3,07	27	**8,28**	326
	25–29	43	97,73	1	2,27	0	0,00	1	**2,27**	44
	30+	13	100,00	0	0,00	0	0,00	0	**0,00**	13
Statistical result *p > 0.05										
Number of still births	0	1068	91,20	61	5,21	42	3,59	103	**8,80**	1171
	1	64	86,49	6	8,11	4	5,41	10	**13,51**	74
	2+	19	95,00	1	5,00	0	0,00	1	**5,00**	20
Statistical result *p > 0.05										
Number of spontaneous abortion	0	753	90,07	47	5,62	36	4,31	83	**9,93**	836
	1	220	91,67	15	6,25	5	2,08	20	**8,33**	240
	2+	179	94,21	6	3,16	5	2,63	11	**5,79**	190
Statistical result *p > 0.05										
Number of living children	0	32	100,00	0	0,00	0	0,00	0	**0,00**	32
	1	118	100,00	0	0,00	0	0,00	0	**0,00**	118
	2	147	90,18	10	6,13	6	3,68	16	**9,82**	163
	3	173	92,02	11	5,85	4	2,13	15	**7,98**	188
	4+	682	89,15	47	6,14	36	4,70	83	**10,85**	765
Statistical result *p < 0.001										
Number of total pregnancies										
	1	96	100,00	0	0,00	0	0,00	0	**0,00**	96
	2	120	100,00	0	0,00	0	0,00	0	**0,00**	120
	3	127	93,38	9	6,62	0	0,00	9	**6,62**	136
	4	104	92,86	6	5,36	2	1,79	8	**7,14**	112
	5+	705	87,91	53	6,61	44	5,49	97	**12,09**	802
Statistical result *p < 0.001										
	**TOTAL**	1152	91,00	68	5,37	46	3,63	114	9,00	1266

*One and two or more induced abortions have been evaluated together in the statistical analyses.

Lifetime induced abortion was found to be significantly higher in women who had got pregnant with their last child without the desire of both of the couples, who wanted no more children and who were using a family planning method (13.31%, 11.95% and 15.21% respectively) (Table 3).

**Table 3:** The number of lifetime induced abortions of women who were ever married and who had at least one pregnancy in their life according to some factors related with family planning

**Number of induced abortions**										
		**0**		**1**		**≥2**		**Women who have made at least one induced abortion**		

		**n**	**%**	**n**	**%**	**n**	**%**	**n**	**%**	Total**

Last pregnancy	Wanted by both of the couples	619	92,39	34	5,07	17	2,54	51	**7,61**	670
	Wanted by only one of the couples	139	91,45	7	4,61	6	3,95	13	**8,55**	152
	Not wanted by both of the couples	306	86,69	25	7,08	22	6,23	47	**13,31**	353
	Total	1064	90,55	66	5,62	45	3,83	111	9,45	1175
Statistical result *p < 0,05										
The state of wanting another child	Wants no more children	707	88,04	55	6,84	41	5,10	96	**11,95**	803
	Wants immediately, wants in the future, undecided	351	95,90	11	3,00	4	1,09	15	**4,09**	366
	Total	1058	90,50	66	5,64	45	3,84	111	**9,49**	1169
Statistical result *p < 0.0001										
Are you using a family planning method	No	596	95,97	17	2,74	8	1,29	25	**4,03**	621
	Yes	485	84,79	49	8,56	38	6,64	87	**15,21**	572
	Total	1081	90,61	66	5,53	46	3,86	112	9,39	1193
Statistical result *p < 0.0001										

*One and two or more induced abortions have been evaluated together in the statistical analyses.

**The evaluations include the ones who have answered the questions.

The lifetime induced abortion rates have been evaluated considering all of the factors thought to be related with induced abortion and has been shown in Tables 1, 2, 3. When we evaluate the results of logistic regression analysis; the number of total pregnancies has been found to be the factor mostly affecting the lifetime induced abortion status (Table 4). Every one point increase of the total number of pregnancies increases the risk of making induced abortion by 1.17 times. The family planning method usage status of the women and the employment status of the women were the other two variables affecting the lifetime induced abortion status of the women. The risk of lifetime induced abortion was found to be 5.4 times greater with women using a family planning method than women not using family planning methods. The lifetime induced abortion risk was found to be 4.1 times greater with unemployed women than working women.

**Table 4:** The results of logistic regression

**Independent Variables**		**Induced Abortion**		
		**p**	**Odds Ratio**	**Confidence Interval (95%)**

				
**Number of total pregnancies**		0,0000	1,17	1,10–1,24
				
**Family planning method**	Not using		1	1
	Using	0,0000	5,35	3,25–8,81
				
**Employment**	Employed		1	1
	Unemployed	0,0000	4,12	2,51–6,77

The rate of induced abortions per 100 lifetime pregnancies – one of the most common indicators of induced abortions – was found to be 2.45. This rate is 1.38 at the rural areas and it rises to 3.33 at the urban areas (p < 0.05) (Table 5).

**Table 5:** The induced abortion rate per 100 lifetime pregnancies among ever married women

		The induced abortion rate per 100 lifetime pregnancies	
**Type of residence**	Rural	1,38	p < 0,05
	Urban	3,33	
	Total	2,45	

"Wanting no more children" is the primary reason given for lifetime induced abortion (64.58%). In 63.54% of the lifetime induced abortions both of the couples have decided to the induced abortion together. Most of the lifetime induced abortions take place at the private doctors' consultant room (46.88%) (Table 6).

**Table 6:** Some characteristics of the women's last induced abortion

		**Rural**		**Urban**		**Total**	
		**n**	**%**	**n**	**%**	**n**	**%**

The reason of the last induced abortion	Wanting no more children	19	65,52	43	64,18	62	64,58
	Short interval between the last two pregnancies	0	0,00	12	17,91	12	12,50
	Mother's health	7	24,14	4	5,97	11	11,46
	Children's health	3	10,34	3	4,48	6	6,25
	The health of mother and children	0	0,00	3	4,48	3	3,13
	Other	0	0,00	2	2,99	2	2,08
							
Who decided to the last induced abortion	Both of the couples together	19	65,52	42	62,69	61	63,54
	Women	2	6,90	18	26,87	20	20,83
	Doctor	7	24,14	5	7,46	12	12,50
	Men	1	3,45	2	2,99	3	3,13
							
Where did the last induced abortion take place	Private doctor	15	51,72	30	44,78	45	46,88
	Public hospital	9	31,03	17	25,37	26	27,08
	Maternity hospital	3	10,34	4	5,97	7	7,29
	Home	1	3,45	6	8,96	7	7,29
	Private hospital/private polyclinic	1	3,45	5	7,46	6	6,25
	Social Insurance Association	0	0,0	4	5,97	4	4,16
	Mother and child health centers	0	0,0	1	1,49	1	1,04
	**Total**	29	100,0	67	100,0	96*	100,0

*96 women have given answer to these questions.

After lifetime induced abortion, 32.29% of the women have not yet begun to use a family planning method. 43.75% of them have since started to use effective methods and 23.96% of them have begun to use ineffective methods. The usage of effective methods was higher in urban areas, while the usage of ineffective methods was higher in rural areas. Intra uterine devices (IUD) (52.38%) took the first and condom (26.19%) took the second place among the effective family planning methods. Withdrawal, with a rate of 87%, took the first sequence among the ineffective family planning methods (Table 7).

**Table 7:** Usage of family planning methods after lifetime induced abortion

Type of residence	**Women using none of the family planning methods**		**Women using an effective family planning method**						**Women using an ineffective family planning method**				**Total**
				
			IUD	Condom	Oral contraceptives	Sterilization of women	**Total effective methods**		Withdrawal	Other ineffective family planning methods	Total ineffective family planning methods		
	
	**n**	**%**	n	n	n	n	n	%	n	n	n	%	n
Rural	11	33,33	5	1	2	-	8	24,24	14	-	14	42,42	33
Urban	20	31,75	17	10	5	2	34	53,97	6	3	9	14,29	63
**Total**	31	**32,29**	22	11	7	2	42	**43,75**	20	3	23	**23,96**	96

In the study, lifetime induced abortions carried out by the women were also evaluated. The number of the women who have stated that "they have tried to make an induced abortion by themselves" in the past was 64. 24 of these women were from rural areas and 40 of them were from urban areas. The women who intended to carry out an induced abortion by themselves firstly preferred to use drugs (43.8%). Lifting heavy things (35.4%) took the second place. Women who live in rural areas preferred to lift heavy things (64.3%) while women in urban areas preferred to take drugs (50.0%).

## Discussion

The percentage of having at least one induced abortion among ever married women who had at least one pregnancy in their life in the SEAP region was 9.0% (lifetime induced abortion rate). Approximately one out of ten ever married women has made at least one induced abortion in their life. Also, 2.45 induced abortion per 100 lifetime pregnancies occurred at the region. When we evaluated the results of the Turkish Demographic and Health Survey 1998; (TDHS 1998) (which is conducted to collect data on subjects such as fertility, infant and child mortality, family planning, and maternal and child health on a representative sample of Turkey through the interviews conducted with women of fertile age) the percentage of lifetime induced abortion among ever married women was reported as 18.2% and induced abortion per 100 pregnancies during the five-year period before the survey was 7.6 for the East Anatolian region (the East Anatolian and the Southeast Anatolian Regions were evaluated together as one region and the SEAP provinces take part in this region). The SEAP rates were lower than the TDHS 1998 [5]. In the TDHS 1998 the lifetime induced abortion rates of the East Anatolian Region were given. The Southeast Anatolian region provinces were evaluated in this region. This study was conducted in the Southeast Anatolian Region only. The general features and health conditions of the Southeast Anatolian Region are worse than the East Anatolian Region, explaining why the rate (9%) is lower than the TDHS 1998.

There is a decrease in the lifetime induced abortion rate in the course of time compared with the TDHS 1998. Also, there is a decrease in the lifetime induced abortion rate in the same region (in the East Anatolian provinces) when the data of the TDHS 1993 is compared with the data of the TDHS 1998. Induced abortion rate per 100 pregnancies during the five-year period before the survey has decreased to 7.6 from 8.7 in the course of time [6,5]. A similar decrease was seen when the Turkey Reproduction Survey-1978 was compared with the TDHS 1998 [7]. In another study conducted in Turkey; abortion rate (both induced and spontaneous abortions) of ever married women was found to be 14.9% in 1991 [8]. In a resent study conducted in Manisa in 2000 induced abortion rate per 100 pregnancies during the five-year period before the survey was found to be 12.1% [9]. It is seen that the induced abortion rate is decreasing not only in the SEAP region but also in other regions of Turkey in the course of time. In a study conducted by Senlet et al. it is reported that there is a decline in induced abortion rates in Turkey [10].

However, this low lifetime induced abortion rates do not show a success because unintended pregnancies end with births in the region. As a matter of fact, 30.1% of the latest births of the women during the last five year period were not desired by both of the couples in the Southeast Anatolian region [11]. Also, total fertility rate of the women was 4.2 in the East Anatolian region [5]. The high fertility rate and the high rate of ending unintended pregnancies with births is the real cause of the low lifetime induced abortion rate in the region.

The rate of induced abortion was higher in urban areas than rural areas. This was similar with the TDHS 1998 [5].

Lifetime induced abortion rate was 7.15% among illiterate women, 10.53% among literate women and was higher among women graduated from primary school or higher (% 12.77, p < 0.05). In a study conducted by Akın et al. similar results have been found [2]. Education is a very important factor effecting induced abortion rate.

In the logistic regression analysis the total number of pregnancies of the women, the family planning method usage status of the women and the employment of the women have been evaluated as the independent factors affecting lifetime induced abortion. As the total number of pregnancies increases, lifetime induced abortion risk increases (odds ratio is 1.7). Women who have more than four children may be the target group of the studies planned on this subject. In a study conducted by Akın et al. a similar odds ratio (1.1) have been found [2].

Lifetime induced abortions were 4.1 times greater with unemployed women than working women. This was due to the fact that these women have lower family planning usage rates but their pregnancy rate was high.

These results remind us that unemployed women and women who have more than four children must be our target group in the campaign against induced abortions as a family planning method.

Lifetime induced abortions were 5.3 times greater with women using a family planning method than women not using family planning methods. I.e. the usage of family planning methods are 5.3 times higher among the women who have had an induced abortion in the past. In a study conducted by Akın et al. similar results were reported during the five-year period before the survey (odds ratio is 2.9) [2].

Lifetime induced abortions have usually taken place at a health facility and with the assistance of health personnel. After these lifetime induced abortions, a qualified family planning consultant can be appointed to these women and they can be encouraged to use a suitable contraceptive method. The rate of effective family planning method usage after induced abortion was 43.7% in our study. The same rate was 34.2% in the TDHS 1998 during the five-year period before the survey [5]. There is an increase in the rate of effective family planning method usage after lifetime induced abortion and this increase is pleasing but it is still insufficient. This increase is thought to be one of the reasons of the decrease in induced abortion rates. Similarly, Senlet and et al has reported that one of the reasons of decrease of the induced abortion rates in Turkey is due to this factor [10].

After lifetime induced abortion, 32.3% of the women were not using a family planning method in the study and this was nearly the same with the percentage evaluated in the TDHS 1998 during the five-year period before the survey (32.1%) [5]. There was no important change during the past four years. In another study in Turkey 25% of the women did not begin to use a family planning method after induced abortion [12]. In two other studies conducted in Turkey it has been found out that approximately 20% of the women did not begin to use a family planning method after induced abortion [13,14]. Also, 23.9% of them have begun to use an ineffective method in our study. These data shows that the family planning services are not adequate at the institutions where induced abortion is performed. Private Doctors (46.88%) and public hospitals (27.08%) were the fist two places where the women applied to have an induced abortion. Similar results have been found in the TDHS 1998 for the Eastern Anatolian provinces during the five-year period before the survey (68.4% and 19.7% respectively) [5]. Similar results were obtained in another study in our country and it has been found out that 50% of the induced abortions were made by private doctors and private doctors were the first place chosen for induced abortion [15]. Thus, priority must be given to educate private gynecologists.

After lifetime induced abortion, 67.71% of the women have begun to use a family planning method in our study.

The primary reason given for the last induced abortion was "wanting no more children" (64.5%) and this is similar with the data of the TDHS 1998 [5]. This is also another indicator for high unintended pregnancy rates and insufficient family planning services in the region. Similar results have been obtained in a different study in our country. In this study 47.6% of the women requested an induced abortion because they wanted no more children [16].

Although the rate of lifetime induced abortions are decreasing in the course of time it is still an important health problem in the SEAP region. Unintended pregnancy and total fertility rates of the region is still higher than the other regions of Turkey. Thus, family planning services, the educational level of women and the status of women need improvement.

## Conclusions

Although 9.0% of the ever married women who had at least one pregnancy in their life have made at least one induced abortion and 2.45 induced abortion per 100 lifetime pregnancies occurred at the SEAP region, these rates are lower than the whole rate of Turkey. But, the high fertility rate shows us that family planning services are insufficient in the region. Also 32.29% have not begun to use a contraceptive method after their last induced abortion and 23.96% of them have begun to use an ineffective contraceptive method. This shows an important lack on this subject. After these lifetime induced abortions a qualified family planning consultant can be appointed to these women and they can be encouraged to use a suitable contraceptive method. Also to decrease lifetime induced abortions; women who have more than four children and unemployed women may be the target group of studies planned on this subject.

## Competing interests

The author(s) declare that they have no competing interests.

## Authors' contributions

AİB participated in the conception and design, provision of study materials, analysis of the data, statistical expertise, drafting the article and revision of the article. BÖ participated in the conception and design, collection and assembly of data, provision of study materials, analysis of the data, statistical expertise, drafting the article, revision of the article and final approval of the article. SÖ participated in the conception and design, collection and assembly of data, provision of study materials, analysis of the data, statistical expertise, drafting the article, revision of the article and final approval of the article. SŞ participated in the conception and design, collection and assembly of data, provision of study materials, analysis of the data, statistical expertise, drafting the article, revision of the article and final approval of the article. TŞ participated in the collection and assembly of data, provision of study materials, analysis of the data and statistical expertise. GS participated in the conception and design, collection and assembly of data, provision of study materials, analysis of the data and statistical expertise. AC participated in the conception and design, collection and assembly of data, provision of study materials, analysis of the data and statistical expertise. Eİ participated in the conception and design, collection and assembly of data, provision of study materials, analysis of the data, statistical expertise, drafting the article, revision of the article and final approval of the article. HA participated in the collection and assembly of data, provision of study materials, analysis of the data and statistical expertise. YP participated in the collection and assembly of data, provision of study materials, analysis of the data and statistical expertise. FA participated in the conception and design, collection and assembly of data, provision of study materials. MA participated in the conception and design, collection and assembly of data, provision of study materials.

## Pre-publication history

The pre-publication history for this paper can be accessed here:


